# Willingness to experience unpleasant thoughts, emotions, and bodily sensations at admission does not predict treatment outcome in inpatients with obsessive–compulsive disorder

**DOI:** 10.1007/s44192-024-00073-6

**Published:** 2024-06-06

**Authors:** Eva M. Zisler, Adrian Meule, Stefan Koch, Ulrich Voderholzer

**Affiliations:** 1 Department of Psychiatry and Psychotherapy, University Hospital, LMU Munich, Munich, Germany; 2 Schoen Clinic Roseneck, Prien am Chiemsee, Germany; 3 Department of Psychology, University of Regensburg, Regensburg, Germany; 4 Medical Center, Department of Psychiatry and Psychotherapy, Faculty of Medicine, University of Freiburg, Freiburg, Germany

**Keywords:** Obsessive–compulsive disorder, Psychotherapy, Exposure and response prevention, Inpatient treatment, Willingness

## Abstract

**Background:**

Some persons with obsessive–compulsive disorder (OCD) refuse or drop out of treatment because of the aversive nature of exposure and response prevention therapy when they have to face and tolerate unpleasant thoughts, emotions, and bodily sensations. Indeed, one study suggested that a higher willingness to experience unpleasant thoughts, emotions, and bodily sensations (WTE) predicts a better treatment outcome, but this finding has not been replicated yet.

**Methods:**

We examined whether WTE at admission predicted treatment outcome in a sample of 324 inpatients with OCD who received a multimodal treatment that included cognitive-behavioral therapy with exposure and response prevention sessions.

**Results:**

Obsessive–compulsive symptoms (based on OCD-specific self-report questionnaires) decreased with medium-to-large effect sizes (all *ps* < 0.001) and global functioning (based on therapist ratings) increased with a large effect size (*d* = 1.3, *p* < 0.001) from admission to discharge. In contrast to previous findings, however, WTE did not predict treatment outcome (all *ps* > 0.005). The effect of WTE on treatment outcome remained non-significant when controlling for any comorbidity, age, sex, length of stay, and antidepressant medication and was not moderated by these variables.

**Conclusions:**

Results indicate that higher WTE at the beginning of inpatient treatment does not facilitate improvements in obsessive–compulsive symptoms from admission to discharge. However, they also indicate that lower WTE at the beginning of inpatient treatment does not adversely affect treatment outcome, that is, even patients who indicate that they are unwilling to face the negative experiences associated with exposure and response prevention can still achieve considerable symptom reductions.

**Supplementary Information:**

The online version contains supplementary material available at 10.1007/s44192-024-00073-6.

## Introduction

Obsessive–compulsive disorder (OCD) is marked by the occurrence of obsessions, compulsions, or both [1]. These can occur in various forms such as unwanted thoughts about aggressive actions, fears of diseases and contamination, or counting compulsions [2]. It is a relatively common mental disorder as the lifetime prevalence ranges between 1 and 3% in the general population [3]. OCD is rarely limited to a single life episode and often has a chronic course when adequate treatment is lacking [1]. Accordingly, many persons with OCD are severely impaired in their daily life and experience substantial distress resulting from their OCD symptomatology [4]. Thus, effective treatment of patients with OCD is of high relevance.

According to international guidelines for the treatment of OCD, psychotherapeutic methods—cognitive-behavioral therapy (CBT) with exposure and response prevention (ERP) in particular—are considered the most effective treatment for OCD [5–7]. This treatment is comprised of systematic exposures to disorder-specific triggers which usually provoke distress, refraining from conducting rituals or avoidance, and cognitive interventions which facilitate learning in exposure sessions [5]. CBT including ERP can be considered very effective in reducing obsessive–compulsive symptoms with large effect sizes of *g* = 1.33 [8].

Despite research providing considerable evidence for the effectiveness of ERP, studies still report dropout rates of 20% in patients with OCD [9, 10]. Amongst others, this might be due to the aversive nature of ERP which challenges the patients to face and tolerate the occurrence of obsessional distress [11]. Hence, in order to successfully undergo ERP, the patient must display high willingness to experience unpleasant thoughts, emotions, and bodily sensations (WTE).

Reid and colleagues [11] investigated 288 adults with OCD receiving residential treatment and assessed self-reported WTE in patients with OCD at three points of measurement: before ERP, immediately after ERP as well as concerning future-exposure WTE. Results showed that higher WTE at all points of measurement was associated with larger symptom reductions. This might be due to several reasons such as high WTE potentially contributing to a reduction in patients’ use of dysfunctional cognitive, behavioral and emotional avoidance strategies during ERP [11]. For example, patients with low WTE may use covert avoidance behaviors (e.g., suppressing thoughts) during ERP, resulting in residual symptoms after successful psychotherapeutic treatment [12, 13]. Furthermore, higher WTE might add to improved extinction learning which is resistant to spontaneous recovery and generalizes to a higher number of stimuli not involved in ERP sessions [11]. Also, there is evidence that high WTE might be linked to mindfulness and enhanced attentional resources as those with increased WTE may have more attentional capacities at their disposal leading to spending less effort in suppressing upcoming thoughts [11]. In sum, the authors thus concluded that WTE may be a clinically relevant marker of ERP response [11].

The current study aimed to replicate the finding by Reid and colleagues [11] that higher WTE predicts better treatment outcome in persons with OCD and extend it to a different sample (which, e.g., also included adolescents and received a longer inpatient treatment than the sample studied by Reid and colleagues). To this end, we analyzed clinical records of persons with OCD who had completed a measure of WTE at admission to treatment. We expected that higher WTE would predict better treatment outcome, that is, larger decreases in obsessive–compulsive symptoms (as measured with self-report questionnaires) and larger increases in global functioning (based on therapist ratings) from admission to discharge.

## Method

### Sample characteristics

Data of inpatients with OCD who were treated at the Schoen Clinic Roseneck (Prien am Chiemsee, Germany) between 2020 and 2022 were analyzed. The treatment provided in the hospital complies with the German S3-guidelines for the treatment of OCD [14]. Therefore, patients received a multimodal treatment that included symptom-specific, individual CBT and ERP sessions, psychotherapeutic group sessions (e.g., based on Acceptance and Commitment Therapy) as well as other treatment elements, depending on indication (e.g., psychopharmacological medication). Data of 324 inpatients with OCD who completed the WTE measure at admission were available. Note that sample size differs for the different analyses because of missing data (Table 1). At the Schoen Clinic Roseneck, data from the diagnostic assessments (e.g., age, sex, diagnoses, medication, length of stay, questionnaire scores) are automatically transferred to a database from which they can be exported without any identifying information by authorized employees. Thus, accessing individual patient charts is not necessary. According to the guidelines by the institutional review board of the LMU Munich, retrospective studies conducted on already available, anonymized data are exempt from requiring ethics approval.

**Table 1:** Descriptive and test statistics for obsessive–compulsive symptoms and global functioning at admission and discharge

*N* = 324	Admission				Discharge				Test statistics		
	*n*	*M*	*SD*	Range	*n*	*M*	*SD*	Range	*Effect size*	*W*	*p*
Obsessive–Compulsive Inventory–Revised											
Washing	322	6.50	4.32	0–12	198	4.01	3.78	0–12	*r*_rb_ = 0.79 (*d* = 0.74)	11410.50	< 0.001
Obsessing	322	8.58	3.13	0–12	198	5.77	3.47	0–12	*r*_rb_ = 0.84 (*d* = 0.92)	14141.00	< 0.001
Hoarding	322	2.88	2.87	0–12	198	2.43	2.69	0–12	*r*_rb_ = 0.34 (*d* = 0.25)	6334.50	< 0.001
Ordering	322	4.98	3.84	0–12	198	3.20	3.30	0–12	*r*_rb_ = 0.77 (*d* = 0.68)	9869.00	< 0.001
Checking	322	6.05	3.78	0–12	198	3.75	3.23	0–12	*r*_rb_ = 0.88 (*d* = 0.88)	12231.00	< 0.001
Neutralizing	322	3.80	4.16	0–12	198	2.38	3.40	0–12	*r*_rb_ = 0.65 (*d* = 0.49)	6479.50	< 0.001
Total Score	322	32.79	12.46	2–69	198	21.53	13.16	1–61	*r*_rb_ = 0.90 (*d* = 1.05)	17737.00	< 0.001
Yale–Brown Obsessive Compulsive Scale											
Obsessions	320	12.42	4.02	0–20	199	8.44	4.11	0–19	*r*_rb_ = 0.82 (*d* = 0.87)	15453.00	< 0.001
Compulsions	320	12.53	4.08	0–20	199	8.23	4.17	0–19	*r*_rb_ = 0.86 (*d* = 0.96)	16166.00	< 0.001
Total Score	320	24.94	6.76	5–39	199	16.66	7.39	0–38	*r*_rb_ = 0.89 (*d* = 1.08)	17490.50	< 0.001
Global Assessment of Functioning	277	44.96	7.07	20–65	277	56.26	9.54	20–98	*r*_rb_ = − 0.97 (*d* = − 1.27)	504.00	< 0.001
Clinical Global Impression—improvement scale	–	–	–	–	278	2.42	0.87	1–6	–	–	–

*r*_rb_ = matched-pairs rank biserial correlation coefficient, *d* = Cohen’s *d*

The majority of patients (79.0%, *n* = 256) had mixed obsessional thoughts and acts (ICD–10 code F42.2), 14.5% (*n* = 47) had predominantly compulsive acts (ICD–10 code F42.1), and 6.5% (*n* = 21) had predominantly obsessional thoughts or ruminations (ICD–10 code F42.0). Mean age was 30.86 years (*SD* = 13.16, Range = 13–70). Two-hundred and seventy-four patients (84.6%) were adults and 50 patients (15.4%) were adolescents. One-hundred and ninety-six patients (60.5%) were female and 128 patients (39.5%) were male. Two-hundred and thirty-eight patients (73.5%) had at least one comorbid mental disorder. The most common comorbid mental disorders were affective disorders (ICD–10 code F3, *n* = 206, 63.3%), anxiety disorders (ICD–10 code F4, *n* = 67, 20.7%), and eating disorders (ICD–10 code F5, *n* = 20, 6.2%). Mean length of stay was 80.84 days (*SD* = 38.55, Range = 2–238). One-hundred and fifty-six patients (56.9%, information missing for 50 patients) received antidepressant medication.

### Measures

*Willingness to experience unpleasant thoughts, emotions, and bodily sensations (WTE).* Similar to Reid and colleagues [11], we assessed WTE at admission with a single question: “How high do you currently rate your willingness to welcome all unpleasant thoughts, feelings, and bodily sensations in the context of planned exposures without avoiding them?”. Responses were recorded on an eleven-point scale from 0 = *very little/little willing* to 10 = *very high/very willing*.

*Obsessive–Compulsive Inventory–Revised (OCI–R).* The German version [15] of the OCI–R [16] was used to measure obsessive–compulsive symptoms at admission and discharge. The OCI–R is an 18-item self-report questionnaire with six subscales: washing, checking, ordering, obsessing, hoarding, and neutralizing. Responses are recorded on a five-point scale ranging from 0 = *not at all* to 4 = *extremely*, referring to the extent of experienced distress during the past month due to OCD symptoms. Internal reliability coefficients for the six subscales and the total scale ranged between ω = 0.77–0.86 at admission and between ω = 0.84–0.90 at discharge.

*Yale–Brown Obsessive Compulsive Scale (Y–BOCS)*. The German version [17] of the Y–BOCS [18] was used to measure OCD severity at admission and discharge. The Y–BOCS is a 10-item self-report questionnaire with two subscales: obsessions and compulsions. Responses are recorded on a five-point scale ranging from 0 = *no symptoms* to 4 = *extreme symptoms*. Internal reliability coefficients for the two subscales as well as the total scale ranged between ω = 0.82–0.86 at admission and between ω = 0.89–0.91 at discharge.

*Global Assessment of Functioning (GAF).* The GAF (American Psychiatric 19) [19]was used to measure patients’ global functioning. Here, therapists rated patients’ global functioning before admission (retrospectively) and at discharge on a scale from 1 = *severely impaired* to 100 = *extremely high functioning*.

*Clinical Global Impression–Improvement Scale (CGI).* The CGI [20] was used to measure change in global functioning during the inpatient stay. Here, therapists rated at discharge if, how much, and in which direction patients’ global functioning changed during treatment on a scale from 1 = *very much improved* and 7 = *very much worse*.

### Data analyses

Data were analyzed with *R* version 4.2.1 [21], *RStudio* version 2022.07.1 [22] and *JASP* version 0.16.4.0 [23]. As some measures were ordinally scaled (WTE, CGI), we used non-parametric and robust techniques for all analyses. Changes in obsessive–compulsive symptoms and global functioning from admission to discharge were tested with Wilcoxon signed-rank tests. Cross-sectional associations between WTE and obsessive–compulsive symptoms and global functioning at admission were examined with robust percentage bend correlation coefficients with the *WRS2* package version 1.1–4 [24, 25].

Longitudinal associations between WTE and treatment outcome measures were tested with robust linear regressions using the *robustbase* package version 0.95–0 [26]. Specifically, separate models were calculated for all treatment outcome measures (i.e., all OCI–R subscale scores and the total score, both Y–BOCS subscale scores and the total score, GAF, CGI) with WTE and the respective admission scores (except CGI, which was only measured once) as independent variables and discharge scores as dependent variable. To examine whether including potential confounding variables affected the longitudinal associations between WTE and treatment outcome, we further ran the same models again while controlling for any comorbidity, age, sex, length of stay, and antidepressant medication. Finally, we also examined whether these variables moderated any longitudinal associations between WTE and treatment outcome by testing interactive effects between WTE and any comorbidity, age, sex, length of stay, and antidepressant medication, respectively, in separate models for each treatment outcome measure and each moderator variable.

For all robust regression models, all continuous variables were *z*-standardized so that all regression coefficients represent standardized coefficients. Because of the numerous inferential tests, we considered effects significant at *p* < 0.005, as has been suggested by others [27]. The data and *R* code with which all robust correlation and robust regression coefficients can be reproduced are available at https://osf.io/rzvuq/.

## Results

Mean WTE ratings at admission were 6.81 (*SD* = 2.53, Range = 0–10). Obsessive–compulsive symptoms decreased and global functioning increased from admission to discharge (Table 1).^1^ WTE was uncorrelated with obsessive–compulsive symptoms and global functioning at admission (Table 2) and did not predict treatment outcome (Table 3).^2^ The effect of WTE on treatment outcome remained non-significant when controlling for any comorbidity, age, sex, length of stay, and antidepressant medication (*bs* = − 0.18–0.03, all *ps* ≥ 0.005) and was not moderated by these variables (interaction effects *bs* = − 0.25–0.29, all *ps* > 0.019).

**Table 2:** Percentage bend correlation coefficients for the relationships of willingness to experience unpleasant thoughts, emotions, and bodily sensations with obsessive–compulsive symptoms and global functioning at admission

	*r*_pb_	99.5% CI	*p*
Obsessive–Compulsive Inventory–Revised			
Washing	0.01	− 0.15; 0.16	0.914
Obsessing	0.005	− 0.15; 0.16	0.932
Hoarding	− 0.10	− 0.25; 0.06	0.073
Ordering	− 0.05	− 0.20; 0.09	0.361
Checking	0.05	− 0.11; 0.21	0.344
Neutralizing	− 0.004	− 0.17; 0.15	0.944
Total Score	− 0.01	− 0.16; 0.15	0.895
Yale–Brown Obsessive Compulsive Scale			
Obsessions	− 0.08	− 0.23; 0.08	0.153
Compulsions	− 0.14	− 0.29; 0.03	0.014
Total score	− 0.12	− 0.27; 0.03	0.028
Global Assessment of Functioning	0.13	− 0.04; 0.29	0.029
Clinical Global Impression–improvement scale	− 0.12	− 0.29; 0.05	0.045

*r*_pb_ = robust percentage bend correlation coefficients

**Table 3:** Standardized coefficients of the robust linear regression models, in which willingness to experience unpleasant thoughts, emotions, and bodily sensations and admission scores were used as independent variables to predict treatment outcome measures at discharge

Dependent variable	Admission scores			Willingness to experience unpleasant thoughts, emotions, and bodily sensations		
	*b*	*SE*	*p*	*b*	*SE*	*p*
Obsessive–Compulsive Inventory–Revised						
Washing	0.70	0.06	< 0.001	0.02	0.06	0.756
Obsessing	0.60	0.05	< 0.001	− 0.15	0.06	0.012
Hoarding	0.72	0.12	< 0.001	− 0.02	0.05	0.726
Ordering	0.70	0.05	< 0.001	− 0.07	0.05	0.216
Checking	0.69	0.05	< 0.001	− 0.11	0.05	0.046
Neutralizing	0.79	0.04	< 0.001	− 0.06	0.05	0.163
Total Score	0.63	0.05	< 0.001	− 0.11	0.06	0.057
Yale–Brown Obsessive Compulsive Scale						
Obsessions	0.44	0.06	< 0.001	− 0.07	0.06	0.273
Compulsions	0.46	0.07	< 0.001	− 0.05	0.07	0.438
Total score	0.45	0.06	< 0.001	− 0.06	0.07	0.328
Global Assessment of Functioning	0.45	0.05	< 0.001	0.04	0.05	0.471
Clinical Global Impression–improvement scale	–	–	–	− 0.09	0.06	0.118

## Discussion

### Summary of results

In the current study, obsessive–compulsive symptoms decreased and global functioning increased with medium-to-large effect sizes, supporting findings about the effectiveness of inpatient treatment for OCD. Yet, WTE at admission to treatment was neither related to obsessive–compulsive symptoms and global functioning cross-sectionally nor related to treatment outcome longitudinally. Controlling for covariates and examining moderators did not change these findings. Our results are in contrast to the findings by Reid and colleagues [11] who found higher WTE to be associated with larger symptom reductions in inpatients after several weeks of treatment. Of note, however, is that we used a conservative threshold for considering effects as significant, suggesting that using a less conservative threshold may have resulted in some significant effects of WTE on treatment outcome. However, when looking at the effect sizes in the current study, it turns out that while the direction of effects was as expected for almost all dependent variables (i.e., higher WTE associated with better treatment outcome), all effects were small (all standardized regression coefficients < 0.2). Therefore, even if WTE significantly relates to treatment outcome in larger samples, it appears that the clinical relevance of this effect may be negligible.

### Clinical implications

This study showed that WTE does not have a significant predictive value for self-reported as well as expert-rated treatment outcome measures in inpatients with OCD. Thus, the results indicate that lower WTE at the beginning of inpatient treatment does not adversely affect treatment outcome. From a clinical point of view, it may be that some patients seem highly motivated to experience unpleasant thoughts, emotions, and bodily sensations during ERP at the beginning of treatment but nevertheless, are unable to fully engage in ERP sessions and still apply some (covert) avoidance behaviors. Furthermore, patients who claim to be unwilling to face negative experiences associated with ERP at admission might still be able to get fully involved in those exercises and achieve considerable symptom reductions. Yet, although the current results indicate that WTE at the beginning of treatment only plays a minor role at most in treating OCD, it may be that the role of WTE increases during treatment. For example, Reid and colleagues [11] who found that higher WTE predicted better treatment outcome assessed WTE multiple times in the course of treatment, suggesting that there may be session-to-session changes in WTE and obsessive–compulsive symptoms which might show a dynamic interplay as therapy progresses.

### Limitations

As in every study, interpretation of the current results is limited to the sample and methods investigated. For example, WTE was measured by a single-item measure. Although using a single-item measure arguably has higher clinical feasibility, future studies might construct a multi-item measure to assess WTE to possibly increase the accuracy of measurement. Furthermore, WTE was assessed based on self-report, which may be biased (e.g., due to demand effects). Thus, future studies are necessary that examine the reliability and validity of self-reported WTE in greater detail, for example, by comparing effects of self-reported WTE with therapist-rated WTE. Another possibility might be to develop a behavioral test for assessing WTE. For example, the Behavioral Avoidance Test (BAT; [31])—which measures how willing a person is to approach symptom-provoking situations or thoughts during OCD-specific tasks—has been found to predict treatment outcome in persons with OCD [28–30]. Future studies might examine whether there is an association between the BAT and self-reported WTE and, thus, whether the BAT may in fact be considered a behavioral measure of WTE. Such studies may then contrast self-reported and behavioral WTE as predictors of treatment outcome.

## Conclusions

In conclusion, results indicate that WTE does not have a significant predictive value for self-report as well as expert-rated treatment outcome measures in inpatients with OCD. This indicates that lower WTE at the beginning of inpatient treatment does not adversely affect treatment outcome which means that even patients who claim to be unwilling to face the negative experiences associated with exposure and response prevention can still achieve considerable symptom reductions. Accordingly, other therapeutic treatment factors may arguably play a greater role than WTE at admission in inpatient treatment [32, 33]. Yet, future studies may examine session-to-session changes in WTE as well as obsessive–compulsive symptoms during treatment which may reveal a dynamic interplay as therapy progresses.

### Supplementary Information

Below is the link to the electronic supplementary material.

Supplementary file 1.

## Data Availability

The data set and annotated *R*-code for our main analyses are available at https://osf.io/rzvuq/.

## Footnotes

1 As some patients had unexpectedly low Y–BOCS scores at admission, we re-ran analyses with patients that had a Y–BOCS score ≥ 18, similar to other studies (e.g., [34]). Yet, results were very similar to those with the full sample (Table S1 in the supplementary material).

2 Again, results were very similar when re-running analyses with the subsample of patients with Y–BOCS scores ≥ 18, that is, WTE did not predict treatment outcome (Table S2 in the supplemental material).
