# Supplementary material for: Willingness to experience unpleasant thoughts, emotions, and bodily sensations at admission does not predict treatment outcome in inpatients with obsessive–compulsive disorder
Source: Discov Ment Health. 2024 Jun 6;4(1):20. doi: 10.1007/s44192-024-00073-6 (PMC11156830; doi:10.1007/s44192-024-00073-6)
Supplement: Supplementary file 1 [file 44192_2024_73_MOESM1_ESM.docx]

Table S1. Descriptive and test statistics for obsessive–compulsive symptoms and global functioning at admission and discharge (exclusion of patients with Y–BOCS values below 18).

| *N* = 275 | Admission | | | |  | Discharge | | | |  | Test statistics | | | |
| --- | --- | --- | --- | --- | --- | --- | --- | --- | --- | --- | --- | --- | --- | --- |
|  | *n* | *M* | *SD* | Range |  | *n* | *M* | *SD* | Range |  | *Effect size* | *W* | *p* |  |
| Obsessive–Compulsive Inventory–Revised |  |  |  |  |  |  |  |  |  |  |  |  |  |  |
| Washing | 273 | 6.99 | 4.19 | 0–12 |  | 165 | 4.36 | 3.89 | 0–12 |  | *r*_rb_ = 0.81 (*d* = 0.77) | 8078.00 | <.001 |  |
| Obsessing | 273 | 8.92 | 2.94 | 0–12 |  | 165 | 6.05 | 3.47 | 0–12 |  | *r*_rb_ = 0.87 (*d* = 0.97) | 9492.00 | <.001 |  |
| Hoarding | 273 | 2.93 | 2.87 | 0–12 |  | 165 | 2.53 | 2.72 | 0–12 |  | *r*_rb_ = 0.32 (*d* = 0.23) | 4392.00 | .003 |  |
| Ordering | 273 | 5.30 | 3.87 | 0–12 |  | 165 | 3.44 | 3.47 | 0–12 |  | *r*_rb_ = 0.78 (*d* = 0.70) | 7342.00 | <.001 |  |
| Checking | 273 | 6.44 | 3.78 | 0–12 |  | 165 | 3.99 | 3.32 | 0–12 |  | *r*_rb_ = 0.86 (*d* = 0.91) | 9597.50 | <.001 |  |
| Neutralizing | 273 | 4.24 | 4.26 | 0–12 |  | 165 | 2.67 | 3.59 | 0–12 |  | *r*_rb_ = 0.68 (*d* = 0.54) | 5033.00 | <.001 |  |
| Total Score | 273 | 34.83 | 12.08 | 5–69 |  | 165 | 23.05 | 13.53 | 1–61 |  | *r*_rb_ = 0.90 (*d* = 1.09) | 12378.50 | <.001 |  |
| Yale–Brown Obsessive–Compulsive Scale |  |  |  |  |  |  |  |  |  |  |  |  |  |  |
| Obsessions | 271 | 13.41 | 3.06 | 3–20 |  | 166 | 8.82 | 4.17 | 0–19 |  | *r*_rb_ = 0.92 (*d* = 1.11) | 11616.00 | <.001 |  |
| Compulsions | 271 | 13.59 | 2.83 | 6–20 |  | 166 | 8.77 | 4.11 | 0–19 |  | *r*_rb_ = 0.93 (*d* = 1.21) | 11981.00 | <.001 |  |
| Total Score | 271 | 27.00 | 4.94 | 18–39 |  | 166 | 17.58 | 7.46 | 0–38 |  | *r*_rb_ = 0.96 (*d* = 1.33) | 12754.00 | <.001 |  |
| Global Assessment of Functioning | 233 | 44.52 | 6.76 | 20–60 |  | 233 | 55.67 | 9.20 | 20–90 |  | *r*_rb_ = −0.96 (*d* = −1.28) | 405.50 | <.001 |  |
| Clinical Global Impression—Improvement Scale | – | – | – | – |  | 234 | 2.44 | 0.87 | 1–6 |  | – | – | – |  |

*Notes*. *r*_rb_ = matched-pairs rank biserial correlation coefficient, *d* = Cohen’s *d*.

Table S2. Standardized coefficients of the robust linear regression models, in which willingness to experience unpleasant thoughts, emotions, and bodily sensations and admission scores were used as independent variables to predict treatment outcome measures at discharge (exclusion of patients with Y–BOCS values below 18).

|  | Admission scores | | |  | Willingness to experience unpleasant thoughts, emotions, and bodily sensations | | |
| --- | --- | --- | --- | --- | --- | --- | --- |
| Dependent variable | *b* | *SE* | *p* |  | *b* | *SE* | *p* |
| Obsessive–Compulsive Inventory–Revised |  |  |  |  |  |  |  |
| Washing | 0.68 | 0.06 | <.001 |  | 0.009 | 0.07 | .895 |
| Obsessing | 0.59 | 0.06 | <.001 |  | −0.15 | 0.07 | .023 |
| Hoarding | 0.71 | 0.11 | <.001 |  | 0.01 | 0.05 | .840 |
| Ordering | 0.70 | 0.06 | <.001 |  | −0.05 | 0.06 | .406 |
| Checking | 0.67 | 0.06 | <.001 |  | −0.11 | 0.06 | .080 |
| Neutralizing | 0.77 | 0.05 | <.001 |  | −0.07 | 0.05 | .169 |
| Total Score | 0.62 | 0.06 | <.001 |  | −0.11 | 0.06 | .095 |
| Yale–Brown Obsessive–Compulsive Scale |  |  |  |  |  |  |  |
| Obsessions | 0.40 | 0.07 | <.001 |  | −0.06 | 0.07 | .400 |
| Compulsions | 0.38 | 0.08 | <.001 |  | −0.05 | 0.07 | .518 |
| Total Score | 0.42 | 0.07 | <.001 |  | −0.05 | 0.07 | .452 |
| Global Assessment of Functioning | 0.43 | 0.06 | <.001 |  | 0.002 | 0.06 | .977 |
| Clinical Global Impression–Improvement Scale | − | − | − |  | −0.07 | 0.07 | .312 |
